# Additional Inhibition of Wnt/β-Catenin Signaling by Metformin in DAA Treatments as a Novel Therapeutic Strategy for HCV-Infected Patients

**DOI:** 10.3390/cells10040790

**Published:** 2021-04-02

**Authors:** Dong Lin, Venu Reddy, Hanadi Osman, Adriana Lopez, Ali Riza Koksal, Sadeq Mutlab Rhadhi, Srikanta Dash, Yucel Aydin

**Affiliations:** Laboratory Medicine and Department of Pathology, Tulane University School of Medicine, New Orleans, LA 70112, USA; vreddy2@tulane.edu (V.R.); hosman1@tulane.edu (H.O.); alopez15@tulane.edu (A.L.); akoksal@tulane.edu (A.R.K.); srhadhi@tulane.edu (S.M.R.); sdash@tulane.edu (S.D.)

**Keywords:** chronic hepatitis C virus (HCV) infection, Wnt/β-catenin signaling, glycogen synthase kinase-3β (GSK-3β), direct-acting antivirus agents (DAA), metformin

## Abstract

Chronic hepatitis C virus (HCV) infection causes hepatocellular carcinoma (HCC). Although HCV clearance has been improved by the advent of direct-acting antiviral agents (DAA), retrospective studies have shown that the risk of subsequent HCC, while considerably decreased compared with active HCV infection, persists after DAA regimens. However, either the mechanisms of how chronic HCV infection causes HCC or the factors responsible for HCC development after viral eradication in patients with DAA treatments remain elusive. We reported an in vitro model of chronic HCV infection and determined Wnt/β-catenin signaling activation due to the inhibition of GSK-3β activity via serine 9 phosphorylation (p-ser9-GSK-3β) leading to stable non-phosphorylated β-catenin. Immunohistochemical staining demonstrated the upregulation of both β-catenin and p-Ser9-GSK-3β in HCV-induced HCC tissues. Chronic HCV infection increased proliferation and colony-forming ability, but knockdown of β-catenin decreased proliferation and increased apoptosis. Unexpectedly, Wnt/β-catenin signaling remained activated in chronic HCV-infected cells after HCV eradication by DAA, but metformin reversed it through PKA/GSK-3β-mediated β-catenin degradation, inhibited colony-forming ability and proliferation, and increased apoptosis, suggesting that DAA therapy in combination with metformin may be a novel therapy to treat HCV-associated HCC where metformin suppresses Wnt/β-catenin signaling for HCV-infected patients.

## 1. Introduction

Chronic hepatitis C virus (HCV) infection is a major risk factor for the development of hepatocellular carcinoma (HCC). Over the past decade, deaths caused by chronic HCV-induced HCC increased by 21.1% [1]. HCV is a positive-sense single-stranded RNA virus with 9.6 kb in length and is unable to integrate into the host genome [2,3]. The HCV genomes encodes a single polyprotein. The polyprotein is processed by cellular and viral proteases to generate 10 polypeptides including three structural proteins (core, E1, and E2), viroprotein p7, and six non-structural proteins (NS2, NS3, NS4A, NS4B, NS5A, and NS5B). The Wnt/β-catenin signaling strongly contributes to tumorigenesis in a variety of tumor types [4,5,6,7,8,9]. β-catenin plays a critical role in the Wnt/β-catenin signaling pathway. Its cellular protein turnover is tightly regulated by ubiquitin-mediated degradation controlled by a phosphorylation-dependent ubiquitylation signal. The stabilization and accumulation of β-catenin is usually induced by aberrant Wnt/β-catenin pathway activation, destruction complex components, or somatic gene mutations of β-catenin [10,11]. Under normal physiological conditions, β-catenin in the cytosol is phosphorylated by GSK-3β and CK1 in the β-catenin destruction complex. The phosphorylated β-catenin undergoes ubiquitination, leading to subsequent proteasomal degradation. Therefore, a low level of cytoplasmic β-catenin is kept under normal physiological conditions. The core protein is a major component of HCV and is not only identified on cytosol but also detected in Golgi apparatus, lipid droplets, and nucleus [12,13]. In the nucleus, it mediates Wnt/β-catenin signaling through increasing the expression levels of Wnt ligands, FZD, and LRP5/6 receptors [14,15], while concomitantly decreasing the transcription of Wnt antagonists SFRP2 and DKK1 [16,17]. Furthermore, due to facilitating the hypermethylation of CDH1 (E-cadherin) gene promoter, the core protein decreases protein expression levels of E-cadherin [18], subsequently less β-catenin is captured by the β-catenin/E-cadherin complexes at the cell membrane, leading to the release of more β-catenin free in the cytosol and an enhanced activation of Wnt/β-catenin signaling. NS5A interacts with the regulatory subunit p85 of PI3K to activate the PI3K/Akt cell survival pathway [19,20]. Akt activation stabilizes β-catenin through the inactivation of GSK-3β and subsequently activates Wnt/β-catenin signaling [21]. In addition, the NS5A protein directly interacts and stabilizes β-catenin [22]. The non-structural proteins NS3/4A, NS5A, and NS5B are important for HCV replication and are thought to be potential therapeutic targets for inhibition by direct-acting antivirus agents (DAA) [23,24]. There are four classes of DAA, which are defined by their mechanisms of actions and therapeutic targets: NS3/4A inhibitors, NS5A inhibitors, and NS5B nucleotide inhibitor and non-nucleoside polymerase inhibitors [25,26]. HCV treatment is dramatically increasing the rates of viral clearance by direct-acting antivirus agents (DAA). Rates of sustained virologic response (SVR) in clinical trials with DAA exceed 95% [27]. However, retrospective studies have shown an unexpectedly high incidence of HCC among HCV-associated patients who received DAA treatments [28,29,30,31,32,33,34,35,36,37,38]. Metformin, an anti-diabetic drug which in recent years has been shown promise as an anticancer drug [39], is an AMP-activated protein kinase (AMPK) activator as well as a protein kinase A (PKA) inhibitor in hepatic cells [40]. Recent studies demonstrated that metformin inhibited proliferation in HCC cells in vitro or in xenotransplantation nude mice [41]. Nevertheless, whether metformin possesses antiproliferative effects on chronic HCV-infected cells after HCV eradication remains elusive.

Here, we reported an in vitro model of cell-based chronic HCV infection and showed that Wnt/β-catenin signaling was activated in chronic HCV infection to promote cell proliferation and colony-forming ability but knockdown of β-catenin decreased cell proliferation and increased apoptosis. Surprisedly, Wnt/β-catenin signaling remained activated despite HCV clearance by DAA, but metformin reversed it by PKA/GSK-3β-mediated β-catenin degradation, inhibited cell proliferation and colony-forming ability, and increased apoptosis.

## 2. Materials and Methods

### 2.1. Cell Culture and HCV Infection

A Huh7.5 cell line was cultured in Dulbecco’s modified Eagle’s medium (DMEM; Life Technologies, Carlsbad, CA, USA) supplemented with 2 mM/L glutamine, sodium pyruvate, nonessential amino acids, 100 U/mL of penicillin, 100 mg/mL of streptomycin, and 10% fetal bovine serum. Huh7.5 cells were infected with JFH-∆V3-EGFP virus (HCV genotype 2a) as previously described [42], and these cells were incubated over 120 days. At the indicated time points, cells were collected for Western blotting and flow cytometry.

### 2.2. Western Blotting

At the indicated time points, cells were collected, washed once with PBS, and resuspended in radioimmunoprecipitation assay (RIPA) buffer for cell lysate preparation. The whole cell lysates were subjected to electrophoresis on 4–12% Bis-Tris gels. After electrophoresis, proteins were transferred to nitrocellulose membranes; after protein transfer, the resulting nitrocellulose membrane was incubated in blocking buffer, washed in Tris-Buffered Saline and Tween 20 (TBST), wrapped with stretch-tite plastic wrap and cut into different strips, each of which covers an arear with a protein of interest located in the middle according to the molecular weight of the prestained marker on the flanking lanes of the gel, across the entire width of the membrane. The different strips were incubated with different primary antibodies including anti-HCV-core (Invitrogen, Waltham, MA, USA, Cat. No. MA−080, diluted 1:1000), HCV-NS3 (Invitrogen, Cat. No. 00117-V, diluted 1:1000), β-catenin (Cell Signaling, Danvers, MA, USA, Cat. No. 9562, diluted 1:1000), c-Myc (Santa Cruz, Cat. No. SC−764, diluted 1:1000), phospho-β-catenin (Cell Signaling, Cat. No. 9561, diluted 1:1000), phospho-GSK-3β (Ser9) (Cell Signaling, Cat. No. 9323, diluted 1:1000), cyclin D1 (Cell Signaling, Cat. No. 2922, diluted 1:1000) and Ki67 (Cell Signaling, Cat. No. 9027, alpha fetoprotein (Abcam, Cambridge, MA, USA, Cat. No. ab133617, diluted 1:1000), and glutamine synthetase (GeneTex, Irvine, CA, USA, Cat. No. GTX630654, diluted 1:1000) antibodies, followed by incubation with a secondary antibody, and visualized by the ECL Western blotting detection system (Amersham, Buckinghamshire, UK).

### 2.3. siRNA Treatment

siRNA specific for β-catenin was purchased (AM51331, silencer validated siRNA, ambion by life technologies, Carlsbad, CA, USA). The single-stranded RNA oligonucleotides were annealed to generate the double-stranded siRNAs. Cells were transfected using lipofectamine RNAiMAX by reverse transfection method in a 6-well plate. For each well, 500 μL Opti-MEM medium was mixed with 20 pmol siRNA against β-catenin or scramble siRNA and 5 μL lipofectamine RNAiMAX. Then, the mixture was incubated for 10–20 min at room temperature. To each well with siRNA-Lipofectamine RNAiMAX complexes, the suspension of 3.5 × 10^5^ cells in 2.5 mL of growth medium without antibiotics was added. After incubation for 2 h at 37 °C, the medium was changed with fresh medium. Cells were harvested for Western blotting, cell counting, and flow cytometry after 4 days.

### 2.4. Quantitative Real-Time Polymerase Chain Reaction (qRT-PCR) Analysis

Total cellular RNA was isolated from uninfected and chronic HCV-infected cells. cDNA was obtained by the High Capacity Reverse Transcription Kit (Applied Biosystems, Waltham, MA, USA) according to the manufacturer’s instructions. The following primers were used:

GAPDH:

5′-CGGAGTCAACGGATTTGGTCGTAT-3′

5′-AGCCTTCTCCATGGTGGTGAAGAC-3′

β-catenin (CTNNB1):

5′-GGUGGUGGUUAAUAAGGCUtt-3′

5′-AGCCUUAUUAACCACCACCtg-3′

All reactions were run using a Bio-Rad iCycler (Bio-Rad Laboratories, Hercules, CA, USA).

### 2.5. Immunohistochemical Staining

We performed immunohistochemical staining using the previously described method [43]. Briefly, slides were deparaffinized for 15 min at 50–60 °C followed by treatment with xylene twice for 5 min. The tissue sections were rehydrated by sequential treatment with 100%, 95%, and 80% alcohol. Peroxidase quenching was carried out by incubation with 3% hydrogen peroxide and 100% methanol for 5 min. The slides were placed in a plastic coplin jar with Reveal Decloaker RTU (Biocare Medical, Pacheco, CA, USA) for 25 min at 95 °C in a steamer for heated antigen retrieval. Following this step, the slides were allowed to cool at room temperature for 20 min. The tissue sections were rinsed in deionized, distilled water and marked using an ImmEdge hydrophobic barrier pen. The slides were incubated with a blocking sniper (Biocare Medical, Pacheco, CA, USA) for 20 min. Immunohistochemical staining of β-catenin, p-ser9-GSK-3β and glutamine synthetase (GS) in HCC tissues and normal liver tissues were examined.

### 2.6. Cell Proliferation Assay

For cell counting, cells were plated at a density of 3 × 10^5^ cells/well in a 6-well plate. Cells were counted after 4 days using EVE Automated Cell Counter. This experiment was performed in triplicate. For MTT (3-(4,5-dimethylthiazol-2-yl)-2,5-diphenyltetrazolium bromide) assay, cells were plated at a density of 7500 cells/well in a 96-well plate in 100 μL of media and cultured for 48 h. Then, 20 μL of 5 mg/mL MTT (MTT was prepared in PBS) was added to each well. Cells were incubated for 3.5 h at 37 °C. Remove media and add 150 μL MTT solvent (4 mM HCl, 0.1% of NP−40 all in isopropanol) to the cells. Cover with tinfoil and agitate cells on an orbital shaker for 15 min. Read absorbance at 595 nm.

### 2.7. Clonogenic Survival Assay

Cells were plated at a density of 7500 cells/well in a 6-well plate and allowed to form colonies for 10 days. Then, the cells were fixed with colony fixation solution (acetic/methanol 1:7) and stained with 0.5% crystal violet solution at room temperature for 2 h. All colonies visible by eye were counted.

### 2.8. HCV Eradication and Metformin Treatment

Chronic HCV-infected Huh7.5 cells were treated with either a combination of two DAA: Ledipasvir (LED) (400 nM) and Sofosbuvir (SOF) (400 nM) or interferon-α (IFN) (1 IU) to eradicate HCV for three rounds. Cells were passaged every 3 days for the first two rounds with the same dose of either DAA or IFN. In the third round of treatment, in addition to DAA or IFN, the cells were with or without metformin treatment for 24 h for both Western blotting analysis and apoptosis analysis with flow cytometry, for 48 h for both cell cycle analysis with flow cytometry and cell proliferation assay with MTT with cell densities of 7500 cells/well in a 96-well plate, and for 10 days for colony-forming ability assay with cell densities of 7500 cells/well in 6-well plate with or without metformin treatment (5 mM).

### 2.9. Statistical Analysis

Error bars in the figures represent the mean and SD of three biological samples. Student’s *t* test was performed to evaluate whether the difference between two conditions was significant. Significant differences were marked with ns *p* > 0.05 * *p* ≤ 0.05 ** *p* ≤ 0.01 *** *p* ≤ 0.001 **** *p* ≤ 0.0001

## 3. Results

### 3.1. In Vitro Model of Cell-Based HCV Long Term Infection System Is Established

**Figure 1:**
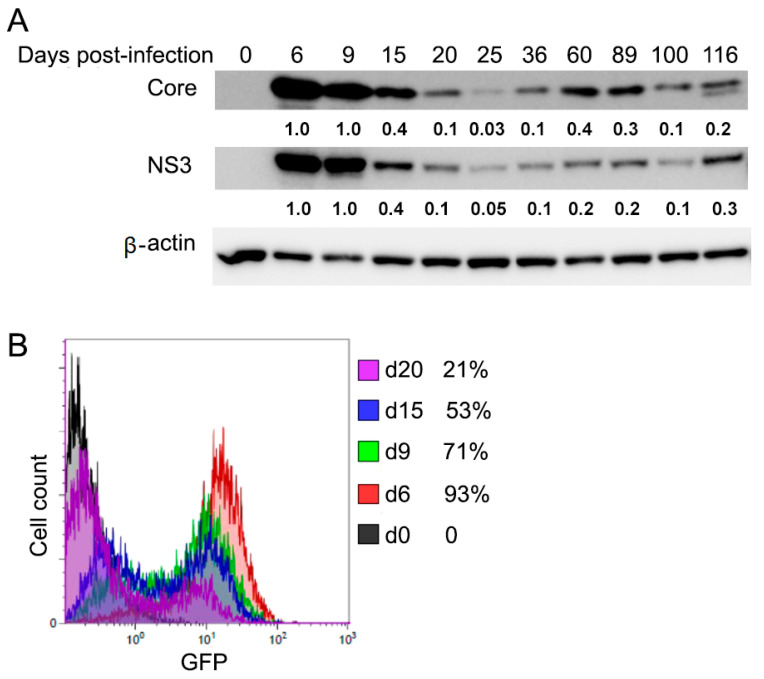
Dynamic expression of hepatitis C virus (HCV) proteins in acute and chronic infection in Huh7.5 cells. (**A**) Cell lysates were taken at the indicated time points (d0, d6, d9, d15, d20, d25, d36, d60, d89, d100, d116) after HCV infection and analyzed for HCV protein Core and NS3 by Western blotting. Quantification of the protein expression levels relative to the β-actin control was expressed as a ratio of the protein expression levels in the cells on day 6 (acute phase) as indicated under each lane. (**B**) Flow cytometry analysis was used to examine GFP-positive populations from HCV-infected cells at the indicated time points.

To characterize the long-term HCV infection in vitro, the cell-based cultivation of HCV was established. Huh7.5 cells were infected with JFH-∆V3-EGFP virus (HCV genotype 2a) at 1 MOI. These cells were incubated over 100 days, which would cover acute and chronic infection and were passaged about 6 days. The infected cells peaked around day 6 (acute phase) as indicated by Western blotting analysis for the HCV core and NS3 protein expressions as well as flow cytometry for HCV-GFP fusion in which 93% of cells were infected, followed by declining production until about day 20 before a chronic phase with fluctuating low-level of production (Figure 1A,B). This experimental data from the current in vitro model of long-term HCV infection exhibited a viral dynamic replication that resembled the patient’s viremia pattern from acute to chronic HCV infection [44,45,46].

### 3.2. Wnt/β-Catenin Signaling Is Activated through Inhibition of GSK-3β Activity in Chronic HCV Infection and HCV-Induced HCC Patient Tissues

**Figure 2:**
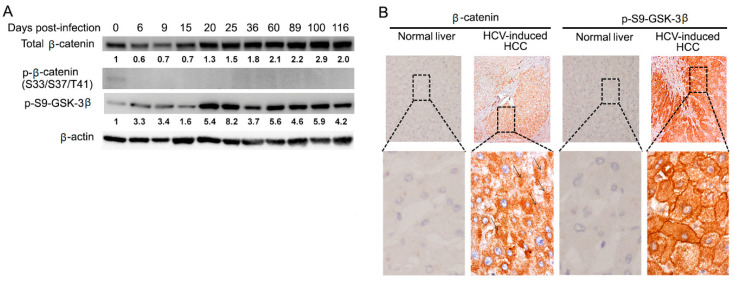
Wnt/β-catenin signaling activation in chronic HC-infected cells and HCV-induced HCC patient tissues. (**A**) Cell lysates were taken at the indicated time points (d0, d6, d9, d15, d20, d25, d36, d60, d89, d100, and d116) after HCV infection and analyzed for total β-catenin, p-β-catenin (S33/S37/T41), and p-ser9-GSK-3β by Western blotting. Quantification of the protein expression levels relative to the β-actin control was expressed as a ratio of the protein expression levels in the uninfected control cells as indicated under each lane. (**B**) Immunohistochemical staining was performed for β-catenin and p-Ser9-GSK-3β in HCV-induced HCC patient tissues.

Dysregulation of Wnt/β-catenin signaling has been suggested to play a critical role in the development of HCC. We hypothesized that Wnt/β-catenin signaling could be involved in chronic HCV infection. We first tested β-catenin protein levels. As indicated in Figure 2A, total β-catenin protein levels increased starting on day 9, although they decrease at the beginning of HCV infection by an unknown mechanism. Therefore, we speculate that the progression of chronic infection is related to the turning point of down-regulation to up-regulation of β-catenin. Then, we investigated the β-catenin mRNA levels by qRT-PCR. Since β-catenin protein levels increased in chronic HCV infection after day 20, we did not test the mRNA levels of β-catenin in different time points, instead of picking up three day points (day 32, 61, and 98) after HCV infection as typical representative of HCV chronic infection. We showed that there was no significant difference between uninfected control and chronic HCV-infected cells (Figure S1). Next, we examined the molecular mechanisms of how β-catenin was stabilized and increased in protein level in chronic HCV infection. One mechanism involved in the stabilization of β-catenin is through the inhibition of GSK-3β activity, which fails to stimulate the phosphorylation of β-catenin, resulting in stabilized non-phosphorylated form of β-catenin. A lack of GSK-3β-mediated phosphorylation on Ser33, Ser37, and Thr41 of β-catenin typically signals resistance to ubiquitin-mediated proteolysis and is thought to be an active β-catenin fraction capable of entering the nucleus to turn on the target genes [47]. To analyze the phosphorylation status of β-catenin, Western blotting was performed with anti-phospho-β-catenin (Ser33/37/Thr41) antibody. As shown in Figure 2A, the signal for phosphorylated β-catenin levels was undetectable in chronic HCV infection, suggesting that the β-catenin protein in chronic HCV infection is the non-phosphorylated form, while it is marginal in uninfected control. Studies indicated that GSK-3β activity is inhibited through the phosphorylation of serine 9 (p-ser9-GSK-3β) by protein kinase A (PKA), Akt (also known as protein kinase B), protein kinase C, p70 S6 kinase, and other kinases [48]. Given the results of an increase of non-phosphorylated β-catenin in chronic HCV infection, we hypothesized that GSK-3β undergoes p-ser9-GSK-3β to inhibit the activity of GSK-3β. As shown in Figure 2A, Western blot analysis demonstrated that p-ser9-GSK-3β expectedly increased. We investigated the expression levels of β-catenin and p-ser9-GSK-3β in surgical specimens of HCV-induced HCC patients. Surgical specimens from 10 cases of HCV-induced HCC patients were collected from the hospital of Tulane University and immunohistochemically stained for β-catenin and p-ser9-GSK-3β. As shown in Figure 2B, we observed that 100% (10 out of 10) of HCV-induced HCC samples showed elevated β-catenin expression compared with the normal liver and that 100% (10 out of 10) overexpressed p-Ser9-GSK-3β in the 10 paired HCV-induced HCC samples. We further examined protein expression levels of glutamine synthetase (GS) and alpha fetoprotein (AFP). Previous studies have shown that activation of the Wnt/β-catenin signaling specifically induces GS expression in liver [49]. We demonstrated that GS protein expression levels were up-regulated in chronic HCV infection (Figure S2A). Immunohistochemistry analysis showed that HCV-induced HCC tissues had an elevated level of GS (Figure S2B). AFP is a fetal-specific glycoprotein that falls rapidly after birth and remains low throughout adulthood. Serum AFP is the most widely used biomarker for early screening of HCC patients [50]. Studies have indicated that a sustained increase in AFP serum level was one of the risk factors of HCC and has been used to identify a high-risk subgroup of HCC [51]. Western blotting analysis showed that AFP expression levels were up-regulated in chronic HCV infection (Figure S2A). Taken together, these results indicated that β-catenin, its upstream regulator p-Ser9-GSK-3β and downstream target gene GS, as well as HCC-related gene AFP, were upregulated in chronic HCV infection and HCV-induced HCC specimens, suggesting that Wnt/β-catenin signaling may play an important role in the development of HCV-induced HCC.

### 3.3. Chronic HCV Infection Increases Cell Proliferation and Colony-Forming Ability

**Figure 3:**
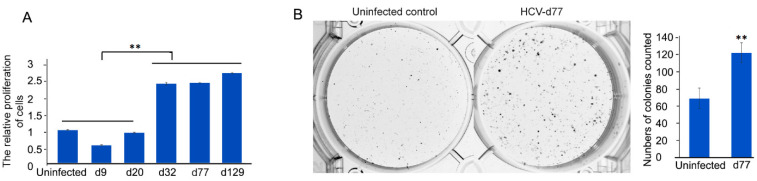
Increased cell proliferation and colony-forming ability in chronic HCV infection. (**A**) HCV-infected cells at the indicated time points post-infection (d9, d20, d32, d77, d129) and uninfected cells were plated at densities of 7500 cells/well in 96-well plates and cultured for 48 h. Cell proliferation was determined by MTT (3-(4,5-dimethylthiazol-2-yl)-2,5-diphenyltetrazolium bromide) assay. Results were calculated on data of triplicate experiments. Graph showed quantification of cell proliferation, ** *p* ≤ 0.01. (**B**) Chronic HCV-infected cells on day 77 (d77) after HCV infection and uninfected cells with the same passage number of chronic infected cells were plated at densities of 7500 cells/well in 6-well plate and cultured for 10 days. The visible colonies were fixed and stained and counted. Graph showed the quantification of colonies (mean ± SD, *n* = 3), ** *p* ≤ 0.01.

To explore the biological consequence of chronic HCV infection, cell proliferation and colony formation ability were examined. Measuring cell proliferation was performed at different time-points with MTT assay. Compared with the uninfected cells, cell proliferation was gradually and dramatically increased over time in chronic HCV-infected cells (more than 20 days post-HCV infection) by 240%, 243%, and 272% at day 32, 77, and 129, respectively, although it was decreased in acute phase (less than 20 days post-HCV infection) by 54% and 92% at day 9 and 20, respectively (Figure 3A). Furthermore, as shown in Figure 3B, the colony-forming assay revealed that the colony-forming ability of chronic HCV-infected cells was significantly higher than that of the uninfected control. The median colony-forming unit (CFU) frequencies were 123.3 of chronic HCV-infected cells vs 69.7 of control in a cell seeding density of 7500 cells/well in a 6-well plate. There was a 77% (123.3 vs. 69.7) increase in colonies of chronic HCV-infected cells in comparison with the control. Taken together, these results suggested that chronic HCV infection promoted both cell proliferation and colony-forming ability.

### 3.4. siRNA-Mediated Knockdown of β-Catenin Inhibits Cell Proliferation through Expanded G1 Phase and Induces Apoptosis

**Figure 4:**
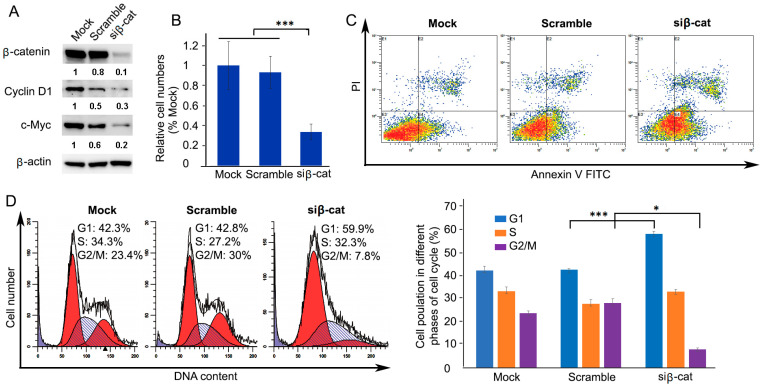
Inhibition of cell proliferation by siRNA-mediated β-catenin knockdown in chronic HCV-infected cells (d84). (**A**) Chronic HCV-infected cells were transiently transfected with β-catenin targeted siRNA for 4 days. Cell lysates were collected for Western blotting with indicated antibodies. Scrambled siRNA was used as the negative control. (**B**) Chronic HCV-infected and uninfected control cells were plated at densities of 3 × 10^5^ cells/well in a 6-well plate and transfected with β-catenin siRNA. Cells were counted after 4 days using EVE Automated Cell Counter. Graph showed quantification of cell counted (mean ± SD, *n* = 3), *** *p* ≤ 0.001. (C) Apoptosis analysis by flow cytometry was performed for the chronic HCV-infected cells transfected with β-catenin siRNA on day 4. (**D**) Cell cycle analysis by flow cytometry was performed for the chronic HCV-infected cells transfected with β-catenin siRNA on day 4. Results are one trial representative of three independent experiments, * *p* ≤ 0.05.

The above data showed that the activation of β-catenin promoted cell proliferation and colony-forming ability in chronic HCV-infected cells. Then, we asked whether knockdown of β-catenin would result in the opposite. To this end, small interfering RNA (siRNA)-mediated knockdown of β-catenin in chronic HCV-infected cells was introduced. Western blotting analysis showed that β-catenin-targeting siRNA significantly decreased protein levels of β-catenin and its downstream gene protein levels of c-Myc and cyclin D1 (Figure 4A). Knockdown of β-catenin resulted in significant inhibition of cell proliferation by 64.3% (1.41 vs. 0.51) at 4 days after transfection of β-catenin-specific siRNA (Figure 4B). Furthermore, cell apoptosis analysis by flow cytometry was performed for the β-catenin-knockdown cells. Figure 4C represents apoptosis events that occurred following β-catenin knockdown compared to the control. Table 1 shows the percentage (%) of distribution of apoptosis events with or without β-catenin knockdown, demonstrating that the percentage of early apoptosis and late apoptosis increased 63.2% (control, 21.63% vs. β-catenin knockdown, 35.3%) and 111.59% (control 6.9% vs. β-catenin knockdown 14.60%), respectively. Non-targeting siRNA (scramble) was included as a negative control and did not alter apoptosis. Cell cycle analysis showed that the proportion of the G1 phase population was significantly increased compared with the scrambled control (scrambled control, 42.9 ± 0.5%, *n* = 3; β-catenin knockdown, 58.7 ± 1.2%, *n* = 3, *p* = 0.0006) resulting in prolonged doubling time, suggesting that cell proliferation was inhibited, while the β-catenin knockdown caused a concomitant decrease in the proportion of G2/M phase population (scrambled control, 28.4 ± 1.6%, *n* = 3; β-catenin knockdown, 8.0 ± 0.6%, *n* = 3, *p* = 0.0018) (Figure 4D). Overall, the results demonstrated that β-catenin knockdown had an antiproliferative effect on chronic HCV-infected cells by activating the induction of apoptotic cells and expanded G1 phase.

### 3.5. Wnt/β-Catenin Signaling Remains Activated in Chronic HCV-Infected Cells Treated by Direct-Acting Antiviral Agents (DAA)

Although HCV have been eradicated, metformin reverses Wnt/β-catenin signaling through PKA/GSK-3β-mediated β-catenin degradation as well as inhibits cell proliferation and colony-forming ability.

**Figure 5:**
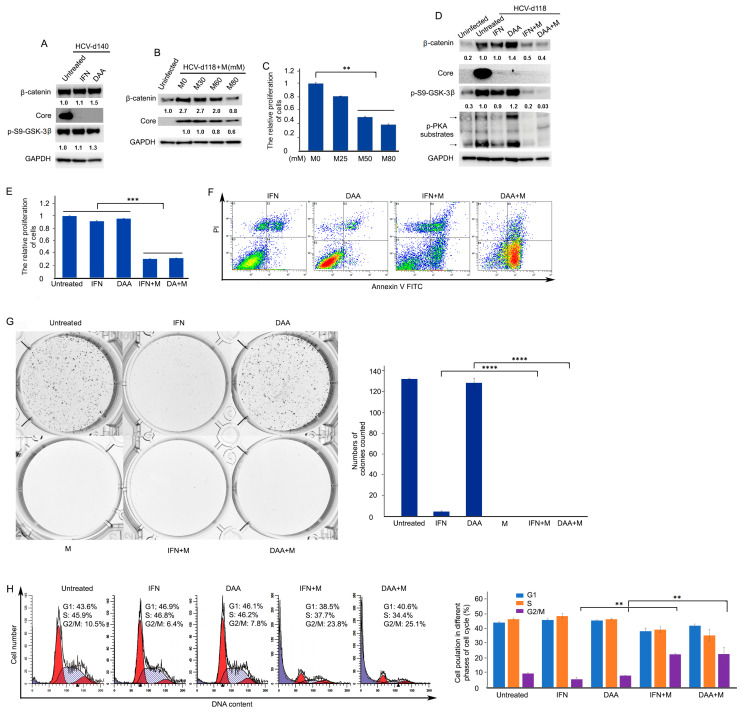
Wnt/β-catenin signaling remains activated after HCV clearance by both IFN and DAA treatments, but it is reversed by metformin through protein kinase A (PKA)/Glycogen synthase kinase (GSK)-3β-mediated β-catenin degradation. (**A**) Chronic HCV-infected cells (day 140) were treated with either a combination of two DAA: Ledipasvir (LED) (400 nM) and Sofosbuvir (SOF) (400 nM) or interferon-α (IFN) (1 IU) to eradicate HCV for three rounds. Cells were passaged every 3 days for each round with the same dose of drugs. After the treatment, cell lysates were collected for Western blotting with indicated antibodies. (**B**) Cells (d118) were treated with different doses of metformin (mM). After 24 h, cell lysates were collected for Western blotting with indicated antibodies. (**C**) Chronic HCV-infected cells (d101) were plated at a density of 7500 cells/well in 96-well plates with different doses of metformin (mM) treatment and cultured for 48 h. Cell proliferation was determined by MTT assay. Results were calculated on data of triplicate experiments. Graph showed the quantification of cell proliferation, ** *p* ≤ 0.01. (**D**) Cells (d118) were treated as above with either DAA or IFN. In the third round of treatment, in addition to either DAA or IFN, the cells were treated with or without metformin (M) (80 mM). After 24 h, cell lysates were collected for Western blotting with indicated antibodies. (**E**) In the third round of treatment as D, cells (d118) were plated at a density of 7500 cells/well in 96-well plates and cultured for 48 h. Cell proliferation was determined by MTT assay. Results were calculated on the data of triplicate experiments. Graph showed the quantification of cell proliferation, *** *p* ≤ 0.001. (**F**) In the third round of treatment as D for 24 h, apoptosis analysis by flow cytometry was performed. (**G**) In the third round of treatment as D, cells (d118) were plated at densities of 7500 cells/well in 6-well plate and cultured for 10 days. The visible colonies were fixed, stained, and counted for the colony-forming ability assay. A graph showed the quantification of cells counted (mean ± SD, *n* = 3), **** *p* ≤ 0.0001. (**H**) In the third round of treatment as D for 48 h, cell cycle analysis by flow cytometry was performed. Results are one trial representative of three independent experiments, ** *p* ≤ 0.01.

**Figure 6:**
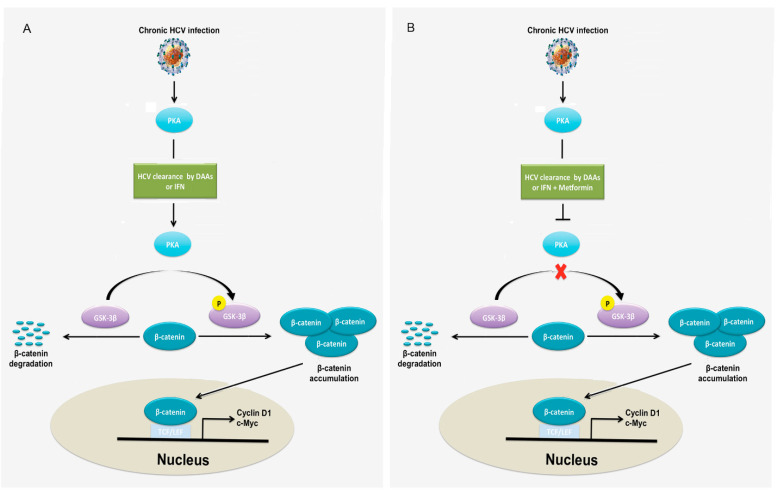
Schematic diagram of the Wnt/β-catenin signaling pathway in chronic HCV infection and HCV clearance without (**A**) or with (**B**) metformin treatment.

Chronic HCV infection is the most significant predisposing factor for HCC. Although the eradication of HCV with DAA seems to be feasible in the majority of patients, it has been shown that there are a measurable number of patients who developed HCC despite viral clearance with DAA regimens [28,29,30,31,32,33,34,35,36,37,38]. However, the factors responsible for HCC development after viral eradication in patients with DAA treatments have not been determined. We hypothesized that Wnt/β-catenin signaling remains activated after HCV eradication by DAA in chronic HCV infection. To verify this hypothesis, chronic HCV-infected cells were treated with either IFN or DAA. Western blotting analysis showed that Wnt/β-catenin signaling remained activated in chronic HCV-infected cells treated by either IFN or DAA because both β-catenin and its upstream regulator p-ser9-GSK-3β protein levels remained high, although HCV were eradicated (Figure 5A). Studies demonstrated that metformin, a drug for diabetes type II, inhibited Wnt/β-catenin signaling in colon cancer cells and lung cancer cells [52,53]. Based on these findings, we asked whether metformin plays a similar role in liver cancer cells. To answer this question, chronic HCV-infected cells were treated with metformin. To assess dose effects, a range (25–80 mM) of doses was used to determine the drug sensitivity of metformin. Our results demonstrated that metformin inhibited β-catenin protein levels and cell proliferation in a dose-dependent manner (Figure 5B,C). It is noticed that metformin also inhibited cell proliferation of uninfected Huh7.5 hepatocellular carcinoma cells (Figure S4). We further examined the effect of metformin on Wnt/β-catenin signaling and analyzed the protein expression levels of components, which are associated with the β-catenin pathway. As indicated in Figure 5D, Western blotting analysis demonstrated that metformin inhibited Wnt/β-catenin signaling after IFN- or DAA-based HCV clearance as indicated by decreased protein levels of β-catenin and its upstream regulator p-ser9-GSK-3β through inhibition of PKA activity, as indicated by decreased phospho-PKA substrates, suggesting that metformin reversed Wnt/β-catenin signaling through PKA/GSK-3β-mediated β-catenin degradation. To further verify the PKA/GSK-3β-mediated β-catenin degradation pathway caused by metformin in chronic HCV infection, we treated chronic HCV-infected cells with PKA inhibitor. As shown in Figure S3, Western blotting analysis indicated that the PKA inhibitor significantly inhibited PKA activity and the protein expression levels of p-ser9-GSK-3β and β-catenin. Taken together, these results suggested that metformin inhibits Wnt/β-catenin signaling through PKA/GSK-3β-mediated β-catenin degradation in chronic HCV infection. The effect of metformin on cell proliferation was studied after HCV eradication. Although IFN and DAA treatments slightly inhibited cell proliferation by 8.7% (untreated, 1 vs. IFN, 0.91) and 4.4% (untreated, 1 vs. DAA, 0.96), metformin treatments in IFN-based and DAA-based HCV-eradicated cells decreased cell proliferation by 66.4% (IFN, 0.91 vs. IFN + M, 0.31; *p* = 0.00077) and 66.7% (DAA, 0.96 vs. DAA + M, 0.32; *p* = 0.00080), respectively, at 48 h of treatment (Figure 5E). Flow cytometry analysis of annexin V-PI labeled cells revealed that metformin increased apoptosis in HCV-eradicated cells by IFN and DAA by 228% (IFN, 15.5 vs. IFN + M, 50.7) and 933% (DAA, 7.9 vs. DAA + M, 81.6), respectively (Figure 5F and Table 2). Moreover, metformin treatments in IFN-based and DAA-based HCV eradication reduced CFU by 100% (IFN, 4.3 vs. IFN + M, 0) and 100% (DAA, 128 vs. DAA + M, 0), respectively at 10 days of treatment (Figure 5G). It is noticed that IFN alone inhibited CFU by 96.7% (untreated, 131.7 vs. IFN, 4.3) while DAA alone slightly inhibited CFU by 2.8% (untreated, 131.7 vs. DAA, 128) compared to the untreated control. We studied the effect of metformin on the cell cycle. The distribution of the cells in the different phases of the cell cycle was analyzed by flow cytometry in HCV-eradicated cells after a 48 h exposure to metformin. Metformin clearly blocked cell cycle progression, inducing an increase in the G2/M fraction in HCV eradication with IFN and DAA by 301.8% (IFN, 5.6 vs. IFN + M, 22.5, *p* = 0.0037) and 180.7% (DAA, 8.1 vs. DAA + M, 22.7, *p* = 0.0038), respectively (Figure 5H), reflecting an arrest of G2/M phase in proliferative cells. Thus, the inhibition of cell proliferation provoked by a 48 h exposure to metformin is mediated by cell cycle arrest in the G2/M phase. Note that the apoptosis induced by metformin in HCV-eradicated cells also contributed to the inhibition of proliferation. Taken together, these results indicated that Wnt/β-catenin signaling remained activated despite HCV clearance, but metformin reversed Wnt/β-catenin signaling through PKA/GSK-3β-mediated β-catenin degradation and inhibited cell proliferation and colony-forming ability and promoted apoptosis (Figure 6).

## 4. Discussion

Cell culture has been employed to grow HCV and provides a feasible in vitro approach for experimental studies of the impact of HCV infection on host cell pathways. Whereas most of the cell culture studies have been used primarily to study the early steps of HCV infection with transient HCV infection for only several days [54], few have considered the long-term persistence (chronic infection) of HCV in cell culture. Furthermore, the impact of chronic HCV infection on the tumor-related pathways of the host cells has not been elucidated. This study established a long-term culture system and characterized the impact of chronic HCV infection on the Wnt/β-catenin pathway of the host cells. We showed that HCV-infected cells peaked around day 6 (acute phase) with 93% of HCV-GFP positive cells, which was followed by a declining production until about day 20 with about 25% of HCV-GFP positive cells and then a chronic phase with a fluctuating low level of production (Figure 1), which resembled the patient’s viremia pattern from acute to chronic HCV infection [44,45]. We determined that Wnt/β-catenin signaling was activated in chronic HCV infection (Figure 2A). The elevated β-catenin protein was due to the inhibition of GSK-3β activity via p-Ser9-GSK-3β. Expectedly, both β-catenin and p-Ser9-GSK-3β were elevated in 100% (10 out of 10) of HCV-induced HCC patient samples (Figure 2B). Our results showed that chronic HCV infection increased cell proliferation over time (Figure 3A) and enhanced colony-forming ability (Figure 3B) but knockdown of β-catenin decreased cell proliferation via an expanded G1 phase and induced apoptosis (Figure 4). We showed for the first time that Wnt/β-catenin signaling activation remained after HCV clearance by either IFN or DAA (Figure 5A). However, metformin reversed the Wnt/β-catenin signaling through PKA/GSK-3β-mediated β-catenin degradation and inhibited cell proliferation and colony-forming ability as well as increased apoptosis (Figure 5B–G). This inhibition of proliferation was confirmed by induction of cell arrest in G2/M phase (Figure 5H). Taken together, these results demonstrated that the β-catenin pathway was activated through the inactivation of GSK-3β in chronic HCV infection to promote both cell proliferation and colony-forming ability but knockdown of β-catenin decreased cell proliferation and induced apoptosis, and that Wnt/β-catenin signaling activation remained after HCV eradication by either IFN or DAA, but metformin reversed it through PKA/GSK-3β-mediated β-catenin degradation and inhibited cell proliferation and colony-forming ability as well as increased apoptosis (Figure 6).

Chronic HCV infection is one of the most significant predisposing factors for HCC. The study and analysis of the HCV genome led to the production of DAA targeting a specific region of its nucleic RNA. DAA is highly effective for the treatment of HCV infection. The SVR exceed 95% in clinical trials, and treatment is well tolerated such that HCV should be considered beyond every doubt a highly curable disease [27]. This high expectation was somewhat shattered by reports of an unexpectedly high incidence of HCC following DAA regimens [28,29,30,31,32,33,34,35,36,37,38]. We indicated for the first time that Wnt/β-catenin signaling remained activated, although HCV were eradiated by DAA (Figure 5A,D), which can be used to explain why patients who received DAA had a high incidence of HCC while HCC was considerably reduced compared with active HCV infection. Furthermore, we showed that the β-catenin protein levels of the cells treated by DAA were higher than those of the cells treated by IFN (Figure 5A,D), which can be used to explain why patients treated with DAA had an apparent increased risk of HCC development when compared with patients treated with IFN [28,34,55].

Given that Wnt/β-catenin signaling remained activated in chronic HCV infection despite virus clearance by DAA, there would be two possibilities for this phenomenon. One possibility is that chronic HCV infection promotes the addition or removal of epigenetic tags on DNA and/or chromatin, leading to a permanent change in the epigenetic profile and/or gene expression of specific genes affected by that epigenetic changes to remain activated Wnt/β-catenin signaling even after HCV clearance [56,57]. The other would be that DAA eradicates HCV and simultaneously activates Wnt/β-catenin signaling. However, from the results that metformin inhibited Wnt/β-catenin signaling (Figure 5B,D), we can exclude the former possibility. Therefore, future study would verify the latter possibility that DAA activates Wnt/β-catenin signaling with uninfected cell lines.

Studies have shown that therapy with metformin was associated with a lower risk of HCC in diabetic patients compared with non-metformin therapy [58,59]. Further studies also have indicated tumor-preventive potential of metformin in non-diabetic patients [60,61]. Recent studies and analysis have shown that metformin reduces the proliferation of cancer cells and the possibility of malignancies in different types of cancer [39]. Metformin is currently the ideal candidate for cancer treatment trials [62]. Studies suggested the effects of metformin on inhibition of mTOR activity through activating ataxia telangiectasia mutated (ATM), liver kinase B1 (LKB1), and AMP-activated protein kinase (AMPK), leading to inhibition of protein synthesis and cell growth [39]. However, the effect of metformin on chronic HCV-infected cells after HCV eradication and the molecular mechanisms involved in regulation of Wnt/β-catenin signaling by metformin remain unknown. Our results indicated that metformin reversed Wnt/β-catenin signaling in either IFN- or DAA-induced HCV eradication through PKA/GSK-3β-mediated β-catenin degradation (Figure 5D). These data provide new insight into how metformin suppressed Wnt/β-catenin signaling following HCV clearance, suggesting a new approach to treatment of HCV-associated HCC with a combination of DAA and metformin so that metformin can suppress Wnt/β-catenin signaling.

However, an in vivo study is needed to further confirm the findings obtained from the in vitro study. The future examination of the liver samples from the HCC patients after HCV clearance by DAA treatment to prove the overexpressed both β-catenin and p-Ser9-GSK-3β is necessary. In the future, clinical trial studies will examine how well DAA with or without metformin works in treating HCV-infected patients. DAA works by blocking viral proteins/enzymes that enable HCV to survive and replicate in host cells. Although our study showed that chronic HCV infection-mediated Wnt/β-catenin signaling remains activated after HCV eradication by DAA, metformin blocked Wnt/β-catenin signaling through PKA/GSK-3β-mediated β-catenin degradation and inhibited cell proliferation and colony-forming ability. Metformin may stop the growth of HCC cells by blocking Wnt/β-catenin signaling. In a clinical trial, we expect that DAA therapy in combination with metformin works better at treating HCV-induced HCC than DAA alone.

This study showed that metformin acts as a PKA inhibitor to activate PKA-mediated GSK-3β activity, leading to PKA/GSK-3β-mediated β-catenin degradation. In fact, besides its role as a PKA inhibitor, metformin also acts as an AMPK activator in human hepatic cells [40]. At this point of off-target effects, in order to more specifically target β-catenin in the future study, an alternative approach is to use RNA-mediated gene silencing, which offers the potential to identify the drug target. RNA interference (RNAi) is a gene silencing system that specifically silences the target gene [63]. RNAi can be exerted through two types of forms: a synthetic small interfering RNA (siRNA) and a vector-based short hairpin RNA (shRNA), that directs the cleavage and degradation of complementary mRNA of target gene. siRNA duplexes are typically transfected into cells for short-term degradation of target molecules (several days), whereas vector-based shRNA can be delivered into cells through expressing vectors for long-term degradation of target molecules. siRNA/shRNA has been widely used to develop more specific therapeutics.

## 5. Conclusions

The Wnt/β-catenin pathway was activated through the inhibition of GSK-3β activity via p-ser9-GSK-3β, leading to stable non-phosphorylated β-catenin accumulation in chronic HCV infection. Immunohistochemistry showed increased both β-catenin and p-Ser9-GSK-3β expression levels in HCV-related HCC tissues. Chronic HCV infection promoted proliferation and colony-forming ability, but siRNA-mediated β-catenin knockdown decreased proliferation and increased apoptosis. Wnt/β-catenin signaling remained activated in chronic HCV infection despite virus clearance by DAA, but metformin inhibited Wnt/β-catenin signaling through PKA/GSK-3β-mediated β-catenin degradation, decreased colony-forming ability and proliferation, and promoted apoptosis. In the future study, we expect that a combination therapy of DAA and metformin may be more efficacious than DAA alone.

## Figures and Tables

**Table 1:** Apoptotic analysis of cells treated by β-catenin-specific small interfering RNA (siRNA) using annexin V-Fluorescein isothiocyanate (FITC) flow cytometry demonstrating viable cells, cells in early apoptosis and late apoptosis.

	Viable Cells (%)	Early Apoptotic Cells (%)	Late Apoptotic Cells (%)
Mock	91.23 ± 0.35	20.50 ± 2.52	6.47 ± 0.21
Scramble	79.47 ± 0.51	21.63 ± 3.17	6.9 ± 0.52
siβ-cat	88.20 ± 0.87	35.30 ± 1.28	14.60 ± 0.85

**Table 2:** Apoptotic analysis of chronic HCV-infected cells treated by either interferon-α (IFN) or direct-acting antiviral agents (DAA) alone and in combination with metformin (M) using annexin V-FITC flow cytometry demonstrating viable cells and cells in early and late apoptosis.

	Viable Cells (%)	Early Apoptotic Cells (%)	Late Apoptotic Cells (%)	Total Apoptotic Cells (%)
IFN	80 ± 1	4.6 ± 0.1	10.9 ± 1.7	15.5 ± 1.8
DAA	89.9 ± 0.9	2.7 ± 0.2	5.2 ± 0.5	7.9 ± 0.7
IFN-M	43.5 ± 1.4	35.9 ± 0.8	14.8 ± 0.3	50.7 ± 1.1
DAA-M	20.1 ± 2.6	70.1 ± 1.8	11.5 ± 0.8	81.6 ± 2.6

## Data Availability

All data presented within this study are available within the manuscript or the Supplementary Materials.

## Supplementary Materials

The following are available online at https://www.mdpi.com/article/10.3390/cells10040790/s1, Figure S1: Quantitative real-time PCR (qRT-PCR) of β-catenin in uninfected and chronic HCV-infected cells, Figure S2: Alpha fetoprotein (AFP) and glutamine synthetase (GS) protein expression levels, Figure S3: Reversed Wnt/β-catenin signaling in chronic HCV infection by PKA inhibitor H89. Figure S4: Metformin inhibited proliferation of un-infected Huh7.5 cells.
