# Supplementary material for: Additional Inhibition of Wnt/β-Catenin Signaling by Metformin in DAA Treatments as a Novel Therapeutic Strategy for HCV-Infected Patients
Source: Cells. 2021 Apr 2;10(4):790. doi: 10.3390/cells10040790 (PMC8065725; doi:10.3390/cells10040790)
Supplement: Supplementary file 1 [file cells-10-00790-s001.pdf]

Supplemental Materials

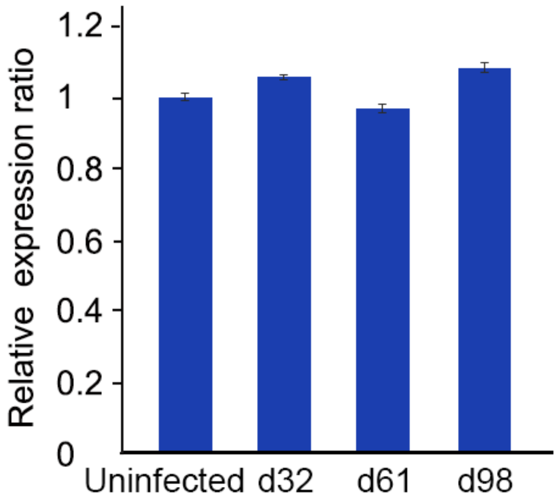

Figure S1

Figure S1. Quantitative real-time PCR (qRT-PCR) of β-catenin in uninfected and chronic HCV-infected cells (d32, d61 and d98). RNAs was isolated from uninfected and chronic HCV-infected cells. Gene expression of β-catenin was analyzed by qRT-PCR.

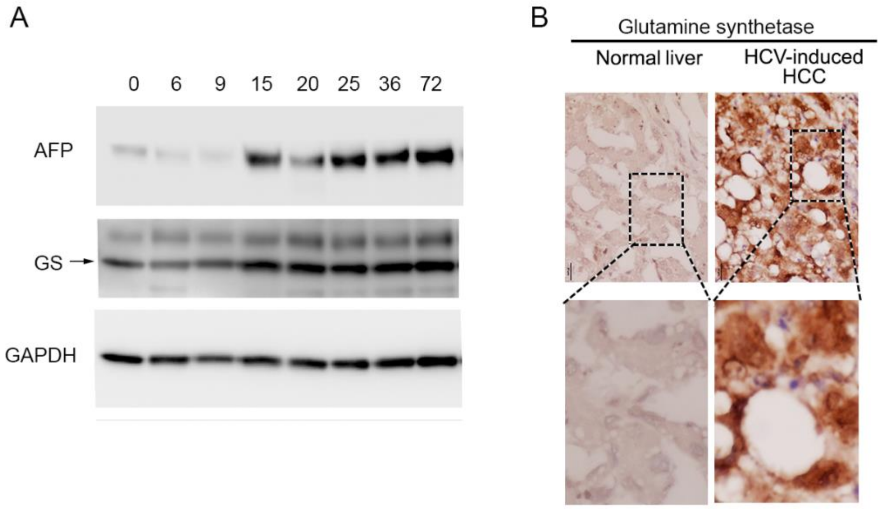

Figure S2

Figure S2. Alpha fetoprotein (AFP) and glutamine synthetase (GS) protein expression levels were upregulated in chronic HC-infected cells and HCV-induced HCC patient tissues. (A) Cell lysates were taken at the indicated time points after HCV infection and analyzed for AFP and GS by western blotting. (B) Immunohistochemical staining was performed for GS in HCV-induced HCC patient tissues.

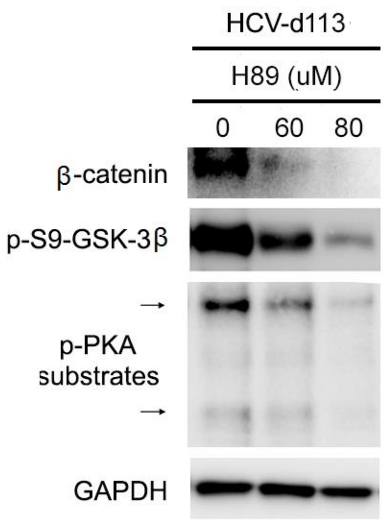

Figure S3

Figure S3. PKA inhibitor H89 reversed Wnt/β-catenin signaling in chronic HCV infection. Chronic HCV-infected cells (d113) were treated with PKA inhibitor H89 for 48 hours. Cell lysates were collected for western blot.

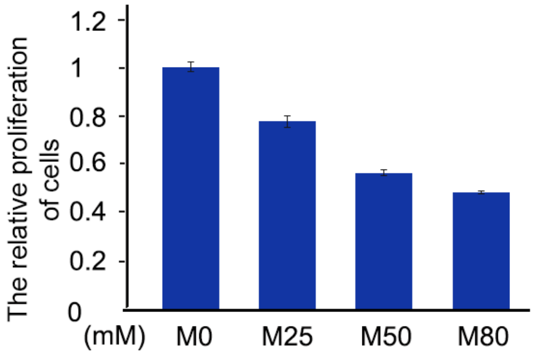

Figure S4

Figure S4. Metformin inhibited proliferation of un-infected Huh7.5 cells. Un-infected Huh7.5 cells were plated at a density of 7500 cells/ well in 96-well plates with different doses of metformin (mM) treatment and cultured for 48 hours. Cell proliferation was determined by MTT assay. Results were calculated on data of triplicate experiments. Graph showed quantification of cell proliferation.
